# Nafcillin-Induced Hepatic Injury: A Case Report and Literature Review

**DOI:** 10.7759/cureus.12817

**Published:** 2021-01-20

**Authors:** Sohaib Khatib, Taher Sabobeh, Michael D Bock, Amgad Masoud, Jwan Alallaf

**Affiliations:** 1 Internal Medicine, University of Missouri Kansas City School of Medicine, Kansas City, USA; 2 Pathology, University of Missouri Kansas City School of Medicine, Kansas City, USA

**Keywords:** nafcillin, drug-induced liver injury, cholestatic, hepatotoxicity, nafcillin-induced liver injury

## Abstract

Background: Drug-induced liver injury (DILI) is the most common cause of acute liver failure in the Western world. While it requires a diagnosis of exclusion, it is exceedingly prevalent in patients taking multiple hepatotoxic agents, the foremost of which are antibiotics, followed by herbal and dietary supplements. Below we will discuss a case of nafcillin-induced liver injury suggested by a thorough work-up and rule-out of other hepatic and biliary pathologies.

Case presentation: We report the case of a 66-year-old white male who presented with painless jaundice. Clinical, laboratory and radiographic features demonstrated a cholestatic pattern of liver injury without significant abnormalities in the biliary tract. All workup for viral hepatitis and autoimmune diseases with liver involvement was negative. Liver biopsy showed acute necro-inflammatory changes suggestive of drug-induced liver injury. The patient had received 18 days of IV nafcillin for blood culture positive methicillin-susceptible *Staphylococcus aureus* (MSSA) four weeks prior to his presentation. He showed clinical and laboratory improvement of his liver functions with supportive care only.

Conclusion: Nafcillin is a safe and effective antibiotic for the treatment of methicillin-susceptible Staphylococcal infections. However, physicians and prescribing healthcare professionals should be aware of the rare, but serious side effects, especially one of drug-induced liver injury with emphasis on the need for early cessation of nafcillin if liver function abnormalities develop.

## Introduction

Drug-induced liver injury is a serious side effect of several drugs; studies have shown that antibiotics are the most common drugs associated with drug-induced hepatotoxicity, with amoxicillin-clavulanic acid being the main one. The other two groups that are also known to cause the majority of drug-induced liver injury are nonsteroidal anti-inflammatory drugs (NSAIDS) and isoniazid [1].

Nafcillin is a beta-lactam antibiotic most commonly used for methicillin-susceptible *Staphylococcus aureus* (MSSA) skin infections. It is also used for the treatment of subacute staphylococcal endocarditis in patients without artificial heart valves [2]. Nafcillin is often prescribed and generally well-tolerated. Drug adverse effects include Clostridioides colitis, oral thrush, and, although uncommon, allergic anaphylaxis [3]. Reports of nafcillin-induced liver injury are rare but serious.

## Case presentation

The patient is a 66-year-old white male with a past medical history of a traumatic brain injury with left-sided hemiplegia since childhood, hypertension, leukemia in remission since 2005, and bilateral renal artery stenosis.

The first time the patient presented to the hospital on day one complaining of generalized weakness and malaise of one week duration. Physical exam was significant for right second toe ulcer with mild erythema and purulence. He had tachycardia on presentation. Laboratory values were significant for white blood cells (WBCs) count of 24.0 and C-reactive protein (CRP) of 13.4. The patient was admitted for infected right second toe with concerns of sepsis. He was treated empirically with intravenous vancomycin and piperacillin-tazobactam. Blood cultures from day two grew methicillin-susceptible Staphylococcus aureus (MSSA) and the patient was switched to intravenous nafcillin 2 g every four hours (12g/day) on day three. Repeat blood cultures on day three grew methicillin-susceptible Staphylococcus aureus (MSSA) and cultures on day seven had no growth. Nafcillin was continued for two weeks following the first negative blood culture. He was discharged to a nursing facility on day 12. He completed 18 days of IV nafcillin on day 21 of his illness.

The patient was re-admitted from the nursing facility on day 20 for a large bloody bowel movement and completed IV nafcillin in the first two days of his second admission. He was worked up appropriately for lower GI bleeding with colonoscopy findings of diverticulosis and recovered with minimal intervention. A liver panel on day 21 was unremarkable: total bilirubin 0.7 mg/dl, alkaline phosphatase (ALK) 60 IU/L, aspartate aminotransferase (AST) 21 U/L, alanine aminotransferase (ALT) 25 U/L.

**Figure 1:**
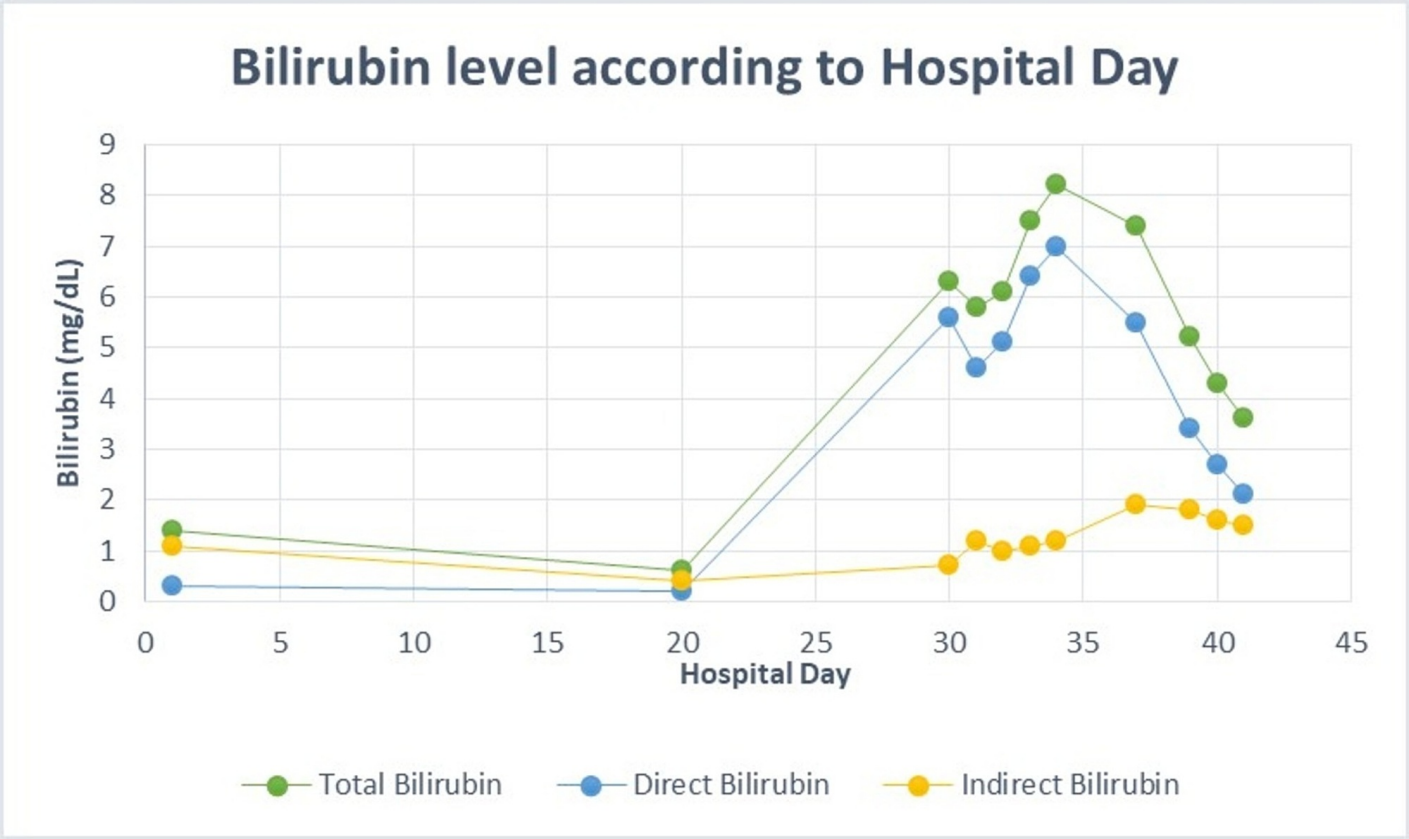
Bilirubin level according to hospital day

**Figure 2:**
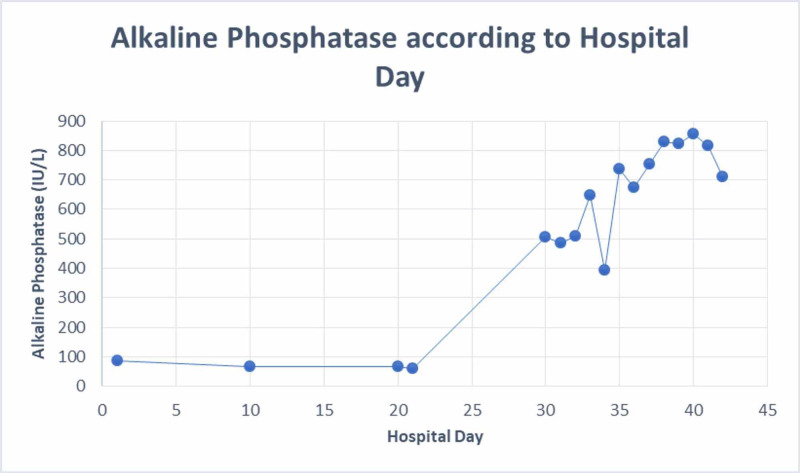
Alkaline Phosphatase according to hospital day

**Figure 3:**
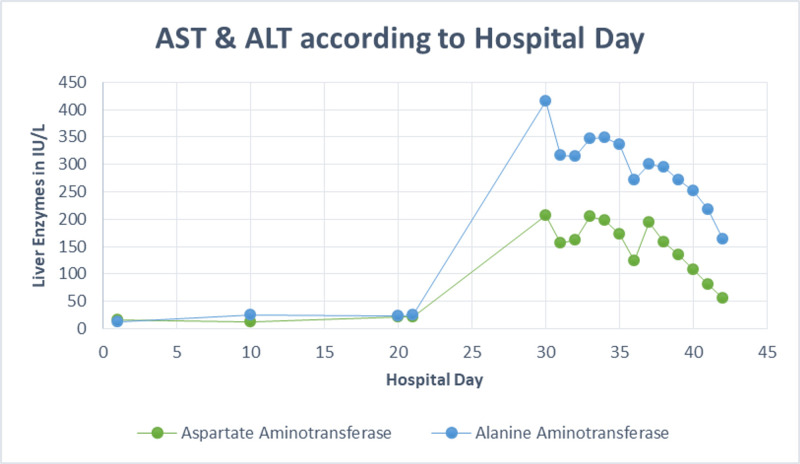
AST and ALT according to hospital day AST: aspartate aminotransferase, ALT: alanine aminotransferase

He was re-admitted for the third time on day 30 of his illness from the nursing home (27 days after IV nafcillin was started and nine days after completion of IV nafcillin) for painless jaundice. Upon re-admission, his labs were remarkable for a cholestasis pattern of liver injury with total bilirubin 6.3 mg/dl (5.6 direct, 0.7 indirect), ALK 506 IU/L, AST 206 U/L, ALT 415 U/L (Figures 1, 2, 3).

**Figure 4:**
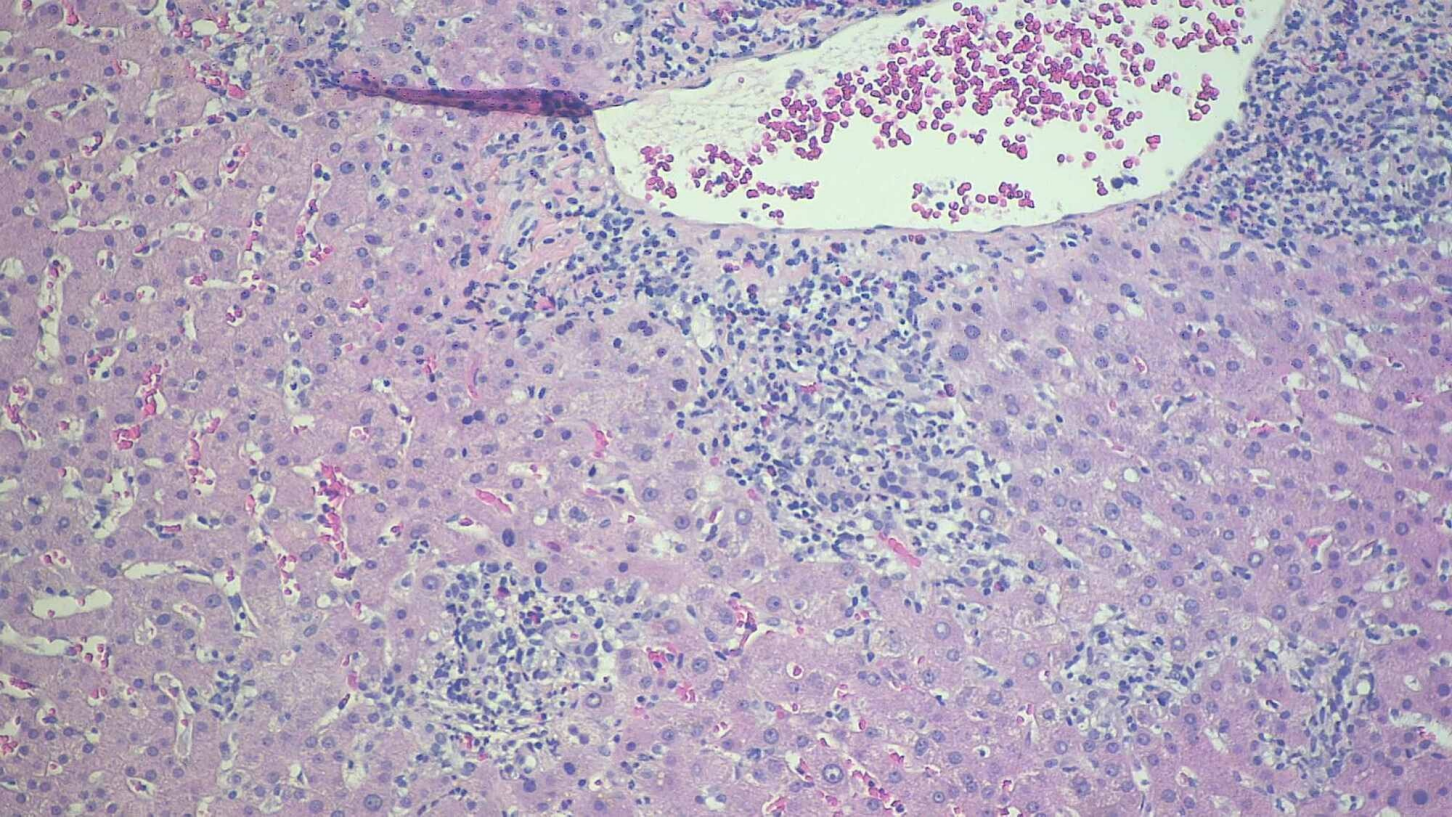
Liver biopsy histopathological examination Sections show lobular necrosis and severe portal tract inflammation with a mixed inflammatory infiltrate of neutrophils, eosinophils, lymphocytes and histiocytes. There is mild bile stasis within hepatocytes. There is no steatosis. PAS and PAS-D are negative for cytoplasmic inclusions. Trichrome stain highlights mild periportal fibrosis.  Prussian blue stain is negative for iron granules .Reticulin stain shows preserved liver architecture.

**Figure 5:**
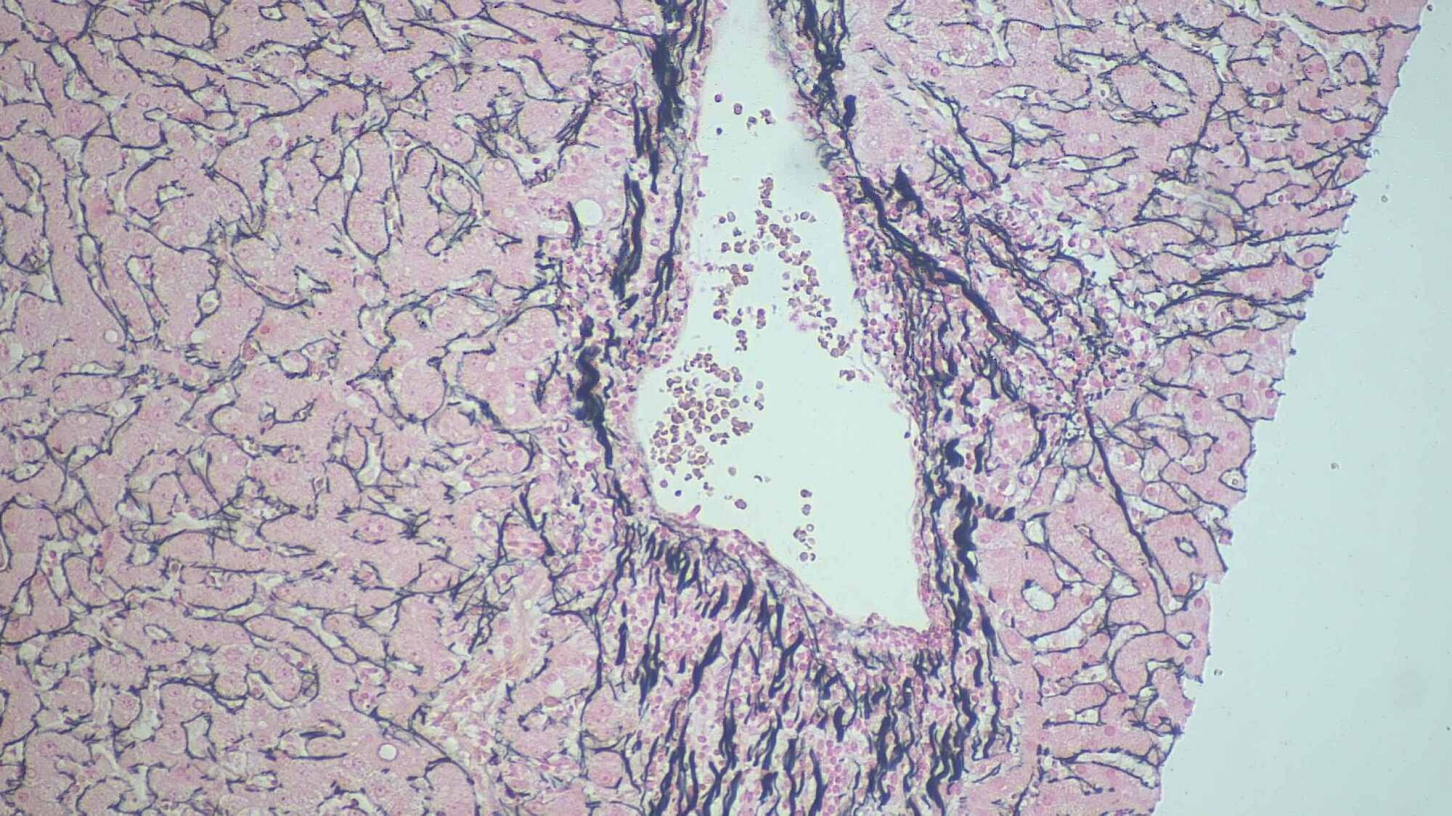
Liver biopsy histopathological examination

**Figure 6:**
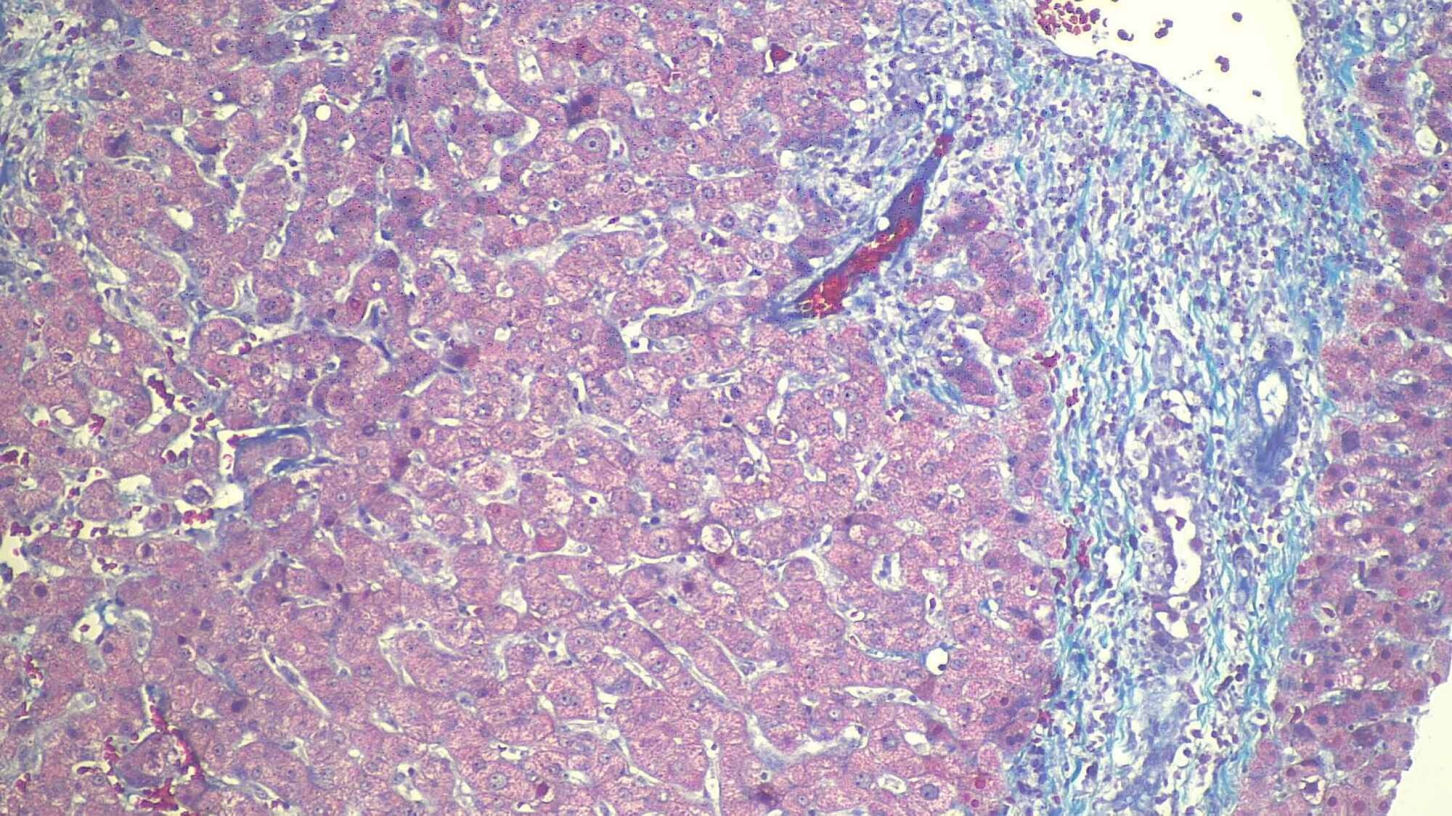
Liver biopsy histopathological examination

At that time his home medications with potential hepatotoxicity including atorvastatin and doxazosin were held. An ultrasound of the liver and biliary tract showed hepatomegaly with normal liver echogenicity, no mass or fluid collection, patent portal and hepatic veins. It also showed cholelithiasis without sonographic signs of cholecystitis, no intrahepatic or extrahepatic biliary ductal dilation. Further workup for infectious causes of liver injury including viral hepatitis panel for hepatitis A, B, C was negative (hepatitis A IgM antibody, hepatitis C antibody, hepatitis B surface antigen, hepatitis B surface and core antibodies all were non-reactive). A serology panel for autoimmune disorders associated with possible liver injury revealed the patient to be negative for anti-nuclear, anti-mitochondrial, anti-smooth muscle, and anti-centromere antibodies. The patient was coronavirus disease 2019 (COVID-19) negative. He does not drink alcohol, smoke cigarettes, or use any recreational drugs. The remainder of the workup was done to exclude alternative liver pathologies. Serum copper and ceruloplasmin, Epstein-Barr virus (EBV) serology, alpha-1-antitripsin all were negative or unremarkable. Magnetic resonance cholangiopancreatography (MRCP) demonstrated cholelithiasis without choledocholithiasis or bile duct dilation. A percutaneous liver biopsy was obtained on day 30 which demonstrated acute necro-inflammatory liver disease suspicious for drug-induced liver injury (Figures 4, 5, 6).

**Figure 7:**
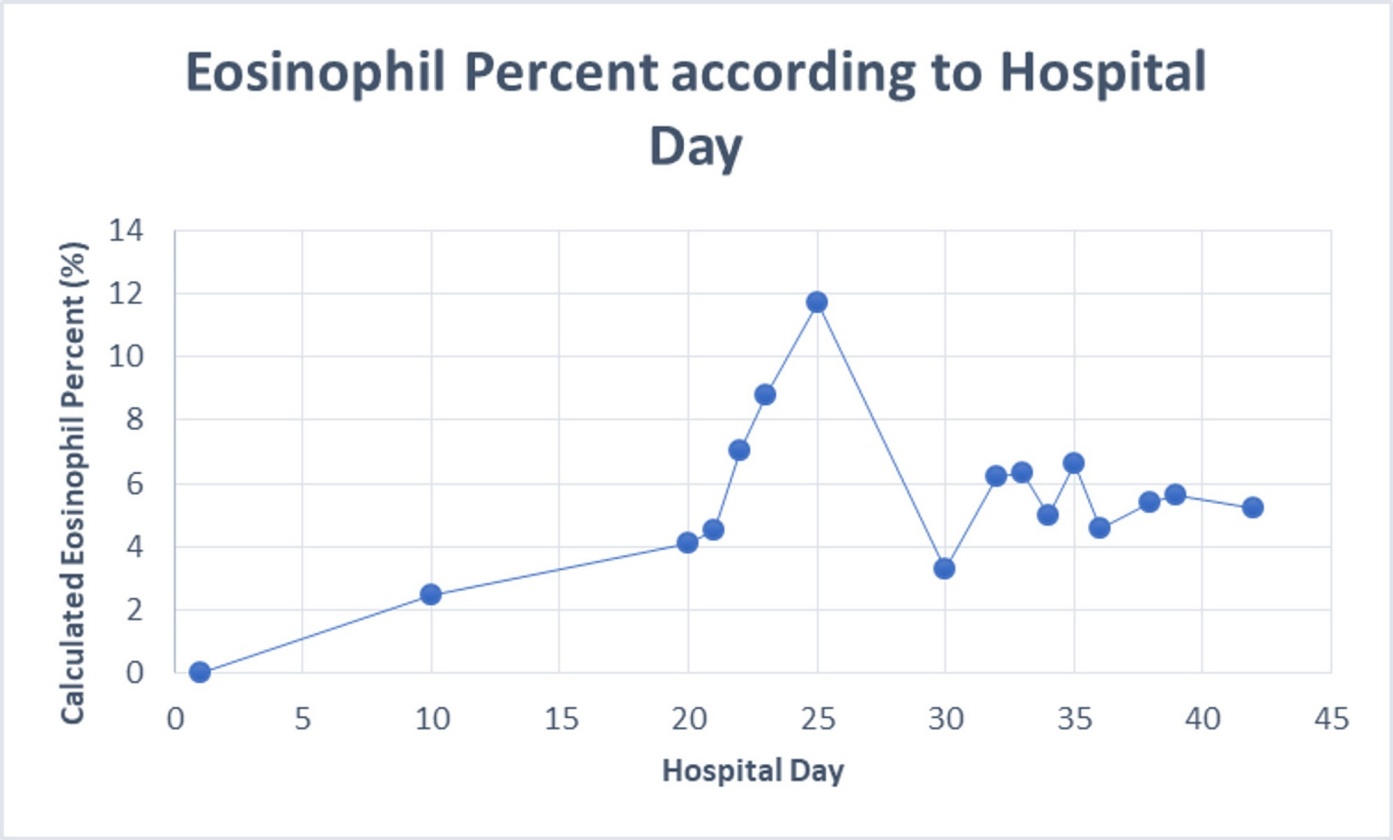
Eosinophil percent according to hospital day

Endoscopic retrograde cholangiopancreatography (ERCP) done on day 33 showed a small amount of biliary sludge that was evacuated after a stent placed in the common bile duct - there was no obstruction or inflammatory changes. A review of prior labs from his previous admission was significant for elevated eosinophil percent on day 25 with 11.7% (normal 1-8%), absolute eosinophil count of 1100 (upper limit of normal is 700) (Figure 7). 

After admission, his liver enzymes continued to worsen, with total bilirubin peaked on day 34 with 8.2 mg/dl (7.0 direct, 1.2 indirect), ALK peaked on day 40 with 865 IU/L, AST and ALT both peaked on day 30 with 206 U/L, 415 U/L, respectively (Figures 1, 2, 3). After ERCP, his liver function tests continued to worsen; the first day total bilirubin started to drop (day 37) was four days after ERCP was done. Throughout his hospital stay, the patient was monitored for signs of encephalopathy including asterixis and mental status changes, which he showed none. In addition to that, international normalized ratio (INR)/prothrombin time test (PT) was monitored daily for coagulopathy but his INR/PT remained within normal limits during his hospitalization. His liver panel steadily declined while he remained in the hospital with supportive treatment only. At time of discharge on day 42, liver lab values were total bilirubin 3.0 mg/dl, ALK 710 IU/L, AST 56 U/L, ALT 164 U/L (Figures 1, 2, 3). See Table 1 for normal laboratory reference ranges. 

**Table 1:** Normal laboratory reference ranges in healthy adult.

Lab test	Normal Lab value range
Aspartate aminotransferase (AST)	(15-41 U/L)
Alanine aminotransferase (ALT)	(7-35 U/L)
Alkaline Phosphatase	(40-129 IU/L)
Total Bilirubin	(0.3-1.2 mg/dl)
Direct Bilirubin	(0.1-0.5 mg/dl)
Indirect Bilirubin	(0-0.7 mg/dl)
Eosinophil percent	(1-8%)
White blood cells count (WBCs)	(4.3-10.8 10^3/cmm)
C-reactive protein (CRP)	(0-1 mg/dl)

## Discussion

Nafcillin is a penicillin antibiotic that is used currently for sensitive staphylococcal infections coverage. Unique about this drug that it is liver excreted through the bile system compared to other antibiotics in the same family (oxacillin and dicloxacillin) [4]. Several cases of nafcillin-induced liver injury have been reported in the literature; some are listed in Table 2 below. 

**Table 2:** Comparison between several case reports of nafcillin-induced hepatotoxicity

Study number/ face of comparison	Baseline Liver function tests (LFTs)	Reason for Nafcillin use	Onset of liver injury	Dose	Outcome	Liver Biopsy	Treatment used	Day of Peak	Pattern of liver injury	Eosinophilia	Reference
1	Normal	Methicillin-susceptible Staphylococcus aureus (MSSA) + blood culture	Day 27	Nafcillin 12 g/day	Complete resolution	Acute necro-inflammatory liver disease	Supportive	33	Cholestatic	Yes (11.7%)	This case
2	Normal	Lumbar Osteomyelitis with methicillin-susceptible Staphylococcus aureus (MSSA)	Day 28	Nafcillin 12 g/day	Complete resolution	Findings consistent with cholestatic hepatitis	Lactulose and empiric IV N-Acetyl Cystine	27	Cholestatic	Yes (20.7%)	[5]
3	Normal	Great toe osteomyelitis with methicillin-susceptible Staphylococcus aureus (MSSA)	Day 15	Nafcillin 12 g/day	Patient died	Picture of cholestatic liver injury	Ursodiol	40	Cholestatic	No	[2]
4	Normal	Culture negative cellulitis	Day 5	Nafcillin 12 g/day	Complete resolution	Findings consistent with cholestatic inflammatory drug reaction	Supportive	45	Cholestatic	No	[6]
5	Not reported	Methicillin-susceptible Staphylococcus aureus (MSSA) septic arthritis	Week 6	Nafcillin, dose not reported	Complete resolution	Findings consistent with cholestatic hepatitis	Supportive	49-55	Cholestatic	Yes (20%)	[4]

Nearly all of the listed cases had cholestatic pattern of drug-induced liver injury. In our case as well, the ALK and bilirubin remained elevated out of proportion to the ALT/AST. The patient in our case showed evidence of immune-mediated idiosyncratic liver injury supported by the finding of eosinophilia (Figure 4); other case reports [4,5] also showed evidence of peripheral eosinophilia (Table 2). This also fit the timeline of weeks to months as opposed to the non-immune pattern which usually takes more than six weeks until onset. [7]

The use of prednisone in drug-induced liver injury is poorly supported due to the lack of sufficient evidence supporting severity reduction and the side effects of long-term use outweighing the potential benefits [8]. Recovery of liver injury in most cases of nafcillin-induced idiosyncratic liver injury occurs between four and twelve weeks. All cases listed in Table 2 including our patient showed complete resolution of liver injury with basically supportive treatment except one case ended with the death of the affected patient secondary to fulminant liver failure [2]. Due to the unusual presentation of nafcillin-induced liver injury, liver biopsy was done in all listed cases in Table 2. The findings were similar with evidence of necrosis, infiltration of several inflammatory cells, and changes consistent of fibrosis. These findings are not specific for drug-induced hepatic injury. However, they keep this diagnosis as one of the most likely differential diagnosis.

In this patient, nafcillin use was terminated prior to onset of elevated liver enzymes. A thorough pharmacy evaluation revealed nafcillin to be the biggest recent change to the patient's medication list. Although atorvastatin and doxazosin were also listed on the patient’s medication reconciliation, they were less likely offenders in this scenario. Atorvastatin had been started many years ago with no recent dose changes. Furthermore, case reports of atorvastatin-induced liver injury favor the hepatocellular pattern [9,10], which includes markedly elevated ALT with lower increases in ALK, unlike our patient. Doxazosin-induced liver toxicity is not only exceedingly rare, but poorly supported by the medical literature, especially to the magnitude seen above [3].

## Conclusions

Nafcillin is an effective and widely used antibiotic at treating staphylococcal infections that are susceptible to methicillin. However, drug-induced liver injury with nafcillin use should not be taken lightly. When prescribing nafcillin for infection, especially in higher doses, a renal and liver panel ought to be evaluated to gauge for idiosyncratic reactions. The best treatment for nafcillin-induced liver injury is to immediately stop the antibiotic, avoid hepatotoxic drugs, and trend the liver enzymes for recovery. In the future, these patients should not be given nafcillin and penicillinase-resistant penicillins, including dicloxacillin and oxacillin.
